# Biotransformation of Methane and Carbon Dioxide Into High-Value Products by Methanotrophs: Current State of Art and Future Prospects

**DOI:** 10.3389/fmicb.2021.636486

**Published:** 2021-03-10

**Authors:** Krishna Kalyani Sahoo, Gargi Goswami, Debasish Das

**Affiliations:** ^1^Department of Biosciences and Bioengineering, Indian Institute of Technology Guwahati, Guwahati, India; ^2^Department of Biotechnology, Gandhi Institute of Technology and Management (GITAM) University, Visakhapatnam, India

**Keywords:** methanotrophs, carbon dioxide, methane, greenhouse gas sequestration, methanol, high-value products, reactor configuration

## Abstract

Conventional chemical methods to transform methane and carbon dioxide into useful chemicals are plagued by the requirement for extreme operating conditions and expensive catalysts. Exploitation of microorganisms as biocatalysts is an attractive alternative to sequester these C1 compounds and convert them into value-added chemicals through their inherent metabolic pathways. Microbial biocatalysts are advantageous over chemical processes as they require mild-operating conditions and do not release any toxic by-products. Methanotrophs are potential cell-factories for synthesizing a wide range of high-value products via utilizing methane as the sole source of carbon and energy, and hence, serve as excellent candidate for methane sequestration. Besides, methanotrophs are capable of capturing carbon dioxide and enzymatically hydrogenating it into methanol, and hence qualify to be suitable candidates for carbon dioxide sequestration. However, large-scale production of value-added products from methanotrophs still presents an overwhelming challenge, due to gas-liquid mass transfer limitations, low solubility of gases in liquid medium and low titer of products. This requires design and engineering of efficient reactors for scale-up of the process. The present review offers an overview of the metabolic architecture of methanotrophs and the range of product portfolio they can offer. Special emphasis is given on methanol biosynthesis as a potential biofuel molecule, through utilization of methane and alternate pathway of carbon dioxide sequestration. In view of the gas-liquid mass transfer and low solubility of gases, the key rate-limiting step in gas fermentation, emphasis is given toward reactor design consideration essential to achieve better process performance.

## Introduction

Emission of greenhouse gases has been increasing globally at an alarming rate. Global anthropogenic emissions of CO_2_ and CH_4_ have almost hit 43.1 billion tons and 9390 mmtCO_2_e, respectively (Global Carbon Project Budget-2019^1^; Global Methane Initiative Report-2018)^2^. The overall global atmospheric concentrations of CO_2_ and CH_4_ have increased by 12.07 and 5.9%, respectively, in the last two decades (NOAA-ESRL, 2020)^3^. Moreover, the global warming potential of CH_4_ is 28–36 times higher than CO_2_ (US-EPA)^4^. There have been efforts to sequester CO_2_ or CH_4_ and catalytically convert them into various value-added products, through hydrogenation and oxidation, respectively. However, chemical processes for the conversion of these C1 compounds are plagued by requirement for expensive catalysts, high temperature (∼450°C), high pressure (∼30 MPa) and release of toxic by-products like carbon monoxide; making the overall technology expensive and non-sustainable.

The use of microorganisms as biocatalysts for the sequestration of CH_4_ and CO_2_ is an attractive alternative as they require milder operating conditions and do not release any toxic by-products. Naturally occurring methane-oxidizing microorganisms are known as methanotrophs. Owing to their ability to utilize methane as the source of carbon and energy, methanotrophs serve as excellent candidates for methane sequestration. Some methanotrophs possess an added advantage of sequestering CO_2_ as substrate for enzymatic hydrogenation into methanol, and thereby qualify as suitable candidates for CO_2_ sequestration. Besides their ability to harness noxious C1 compounds, methanotrophs can also serve as potential cell-factories for a wide-range of high-value products, e.g., methanol, ectoine/hydroxyectoine, poly-β-hydroxybutyrate (PHB), single cell protein, extracellular polysaccharide, lipids etc. (Xin et al., 2007; Rostkowski et al., 2013; Cantera et al., 2018; Rasouli et al., 2018; Tsapekos et al., 2020). Despite their enormous potential, large-scale production of these high-value products is constrained by various limitations in solubility of gases in liquid medium and gas-liquid mass transfer, eventually resulting in -insignificant product titer. The present review provides an overview of methanotrophs, metabolic pathways to utilize CH_4_/CO_2_ and the high-value products synthesized by them. This review also sheds light on the biological production of methanol, a key product targeted as solvent and biofuel, through oxidation of CH_4_ and alternatively via reduction of CO_2_. In view of overcoming the rate-limiting factors in gas fermentation, toward efficient capture and conversion of harmful C1 compounds into high-value products at industrial-scale, special emphasis has been given on reactor design and configuration.

## Methanotrophs

Methanotrophs are Gram-negative proteobacteria, noted for their ability to utilize methane as the sole source of carbon and energy. Methanotrophic bacterium was discovered by Söhngen in 1906 (Hanson and Hanson, 1996). Whittenbury et al. (1970) carried out comprehensive isolation and characterization of methanotrophs and introduced the Type I, Type II, and Type X classification system. Methanotrophs utilize methane via a metabolic cascade comprising of four enzymes, namely, methane monooxygenase (MMO), methanol dehydrogenase (MDH), formaldehyde dehydrogenase (FADH), and formate dehydrogenase (FDH) (Xin et al., 2004a, 2007). Based on the type of MMO produced, the methanotrophs are divided into three categories namely, (a) Type I (produce particulate MMO), (b) Type II (produce both particulate as well as soluble MMO) and (c) Type X (comprises of specific features of both Type I and Type II methanotrophs).

Aerobic methanotrophs were later on classified broadly into two major groups of proteobacteria based on 16S rRNA gene sequencing, namely, gamma-proteobacteria (Group I) and alpha-proteobacteria (Group II), in place of the earlier 3-type classification (Ge et al., 2014). Gamma-proteobacteria (Group I) comprise of previously classified Type I and Type X methanotrophs and Alpha-proteobacteria (Group II) comprise of formerly known Type II methanotrophs (Fei et al., 2014). The sub-division alpha-proteobacteria consists of four genera namely, *Methylocella*, *Methylocapsa*, *Methylocystis*, and *Methylosinus*. The sub-division gamma-proteobacteria consists of 12 genera namely, *Methylothermus, Methylosoma, Methylosphaera, Methylosarcina, Methylomonas, Methylohalobius, Methylomicrobium, Methylococcus, Methylocaldum, Methylobacter, Clonothrix*, and *Crenothrix* (Ge et al., 2014). Gamma-proteobacteria (Type I and Type X) and alpha-proteobacteria (Type II) use ribulose monophosphate (RuMP) cycle and serine cycle, respectively, for utilization of C1-carbon sources, such as formaldehyde/formate. Although, Type X species (*Methylocaldum* and *Methylococcus*), also utilize formaldehyde through RuMP cycle, yet they differ from Type I species as they express small quantities of enzymes of the serine cycle, ribulose-bisphosphate carboxylase, present in the Calvin-Benson-Bassham (CBB) cycle (Hanson and Hanson, 1996; Park and Kim, 2019). Type X species reportedly possess genes encoding enzymes for CO_2_-fixation through CBB cycle (Baxter et al., 2002). Novel methanotrophs have also been found in the phylum Verrumicrobia, which has been reported to fix CO_2_ by utilizing CBB cycle (Rasigraf et al., 2014).

Gamma-proteobacterial and alpha-proteobacterial methanotrophs can also be distinguished based on few other characteristics. In terms of arrangement of their intracytoplasmic membranes (ICMs), gamma-proteobacterial species contain bundles of ICMs, whereas, ICMs in alpha-proteobacterial species are aligned around the cell’s periphery (Kalyuzhnaya et al., 2019). As per cellular phospholipid fatty acid (PLFA) composition, Type I, Type II, and Type X methanotrophs, respectively, possess 14− 16−, 18−, and 16-carbon long PLFA (Ge et al., 2014). On the basis of storage carbon, most species of gamma-proteobacteria are known for glycogen accumulation and a few species for PHB accumulation; while, alpha-proteobacterial species are predicted to mainly accumulate PHB, since they excrete acetone, succinate, acetate, etc. (which are probable derivatives of PHB) from methane fermentation (Kalyuzhnaya, 2016).

Typical growth rates for gamma-proteobacterial and alpha-proteobacterial methanotrophs ranges within 0.009–0.4 and 0.005–0.16 h^–1^, respectively (Kalyuzhnaya, 2016). Methanotrophs are resistant to desiccation. They have been reported for bioremediation, bioleaching, and epoxidation. Conjugation (Nguyen et al., 2020) and electroporation-based (Yan et al., 2016) gene-transfer techniques have been developed for methanotrophs. However, the yield and productivity from methanotrophic cell factories are relatively low as compared to other hosts such as *E. coli* (Lee et al., 2016). Moreover, their obligate methanotrophic characteristics and slow growth rates limits the application of genetic engineering techniques. On the contrary, their obligate methanotrophic nature makes them a cheap industrial platform which can sequester waste greenhouse gas (methane), relative to other platforms which consume expensive substrates like glucose, xylose etc.

Few studies have explored genome-scale metabolic models (GSMM) for methanotrophs for extensively modeling the entire array of genes, metabolites and biochemical reactions *in silico*. Gupta et al. (2019) built a GSMM for *Methylococcus capsulatus* (Bath) comprising of 865 metabolites, 899 reactions and 535 genes. The model could predict the pathways and amino acids necessary for growth, and identified essential metabolic genes and lethal genes in the bacterium. Lieven et al. (2018) using GSMM predicted that methane oxidation by pMMO can be stimulated either through uphill electron transfer or direct-coupling, at decreased efficiency. GSMM helps the authors to build a centralized knowledge-base for the model organism, understand its metabolic physiology and simulate its metabolic behavior under diverse conditions.

## Metabolic Pathway Toward Utilization of CH_4_ and CO_2_

**FIGURE 1:**
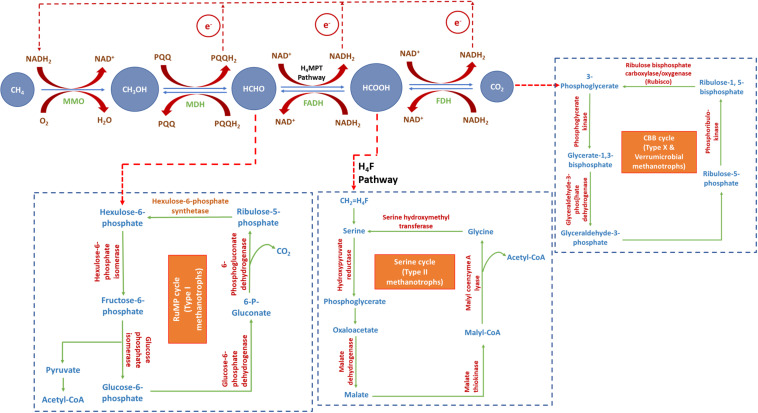
Metabolism of CH_4_ and CO_2_ in methanotrophs (modified from Xin et al., 2007; Park and Kim, 2019). MMO, methane monooxygenase; MDH, methanol dehydrogenase; FADH, formaldehyde dehydrogenase; FDH, formate dehydrogenase.

All aerobic methanotrophs oxidize CH_4_ to CO_2_ through a common enzymatic cascade. The oxidation process produces methanol, formaldehyde and formate as reaction intermediates (Figure 1). This is accomplished through sequential catalysis by the enzymes, MMO, MDH, FADH, and FDH. A fraction of the formate/formaldehyde, produced as an intermediate, gets incorporated into the biomass as a source of carbon through the serine cycle or RuMP cycle. RuMP cycle involves assimilation of formaldehyde and its further conversion into hexulose-6-phosphate. Successive conversions produce ribulose-5-phosphate, thus closing the cycle. Formaldehyde gets transformed into formate through H_4_MPT pathway. Serine cycle involves assimilation of formate. Formate is transferred into serine cycle through H_4_F pathway. Successive intermediate reactions convert serine into glycine, closing the cycle. Assimilation of CO_2_ in Type X and Verrumicrobial methanotrophs via CBB cycle begins with the formation of 3-phospho-glycerate, which eventually gets converted into ribulose-1,5-bisphosphate, completing the cycle (Park and Kim, 2019).

Methane is oxidized to methanol by MMO, which requires two units of reducing equivalents (NADH) to break the double bond in oxygen. One of these oxygen atoms oxidizes methane into methanol, whereas the other gets hydrogenated to produce water. The oxidation of methanol to formaldehyde releases PQQH_2_ and the oxidation of formaldehyde to CO_2_ via formate releases NADH. NADH thus generated is reutilized by MMO for the oxidation of methane to methanol (Xin et al., 2007).

MMO is the key enzyme responsible toward growth of methanotrophs since these microbes utilize methane as the primary source of carbon and energy. MMO is of two types, soluble MMO (sMMO) and particulate MMO (pMMO). sMMO is synthesized in the cytoplasm and is made up of two monomeric units. Each sMMO monomer consists of two iron atoms (Rosenzweig et al., 1995). pMMO is impregnated on the membrane, and is composed of three monomeric units with each unit comprising of either a di-copper or a mono-copper center (Culpepper and Rosenzweig, 2012). Therefore, concentrations of iron and copper ions primarily affect the MMO activity in culture media. High concentration of iron ions is known to stimulate sMMO expression (Chidambarampadmavathy et al., 2015). Additionally, sMMO is actively expressed at lower ratios of copper to biomass, and its expression is repressed at higher concentrations of copper in the medium (Park et al., 1991). On the contrary, pMMO has increased levels of expression at higher ratios of copper to biomass, and hence corroborates improved activity with increase in copper concentration in the medium (Park et al., 1992). Since pMMO is integrated into the membrane, it is supposed to rapidly oxidize methane compared to sMMO. However, authors have outlined that sMMO causes rapid oxidation of methane as compared to pMMO (Sirajuddin and Rosenzweig, 2015). The concentrations of these metal ions significantly influence MMO activity. Higher cellular MMO activity is associated with higher NADH generation (Zhang et al., 2008), which is a necessary cofactor for formation of various methanotrophic products. Few studies have reported the effect of Cu^2+^ and Fe^2+^ ions on the production of PHB and methanol by methanotrophs (Zhang et al., 2008; Patel et al., 2016b).

Some methanotrophs can also utilize CO_2_ as substrate and reduce it to methanol, in presence of excess CO_2_ (Xin et al., 2007). This happens in a two-stage process, where methane is utilized as carbon substrate during the first-stage to produce biomass, and CO_2_ is reduced by the generated biomass in the second-stage to produce methanol. CO_2_-reduction to methanol is the reverse reaction of methane-oxidation to CO_2_. It is a sequential catalysis carried out by FDH, FADH and MDH (Figure 1). Since MMO lacks the ability to catalyze this reverse reaction, the end-product of the CO_2_ reduction pathway is methanol (Xin et al., 2004a). The reduction of CO_2_ to methanol requires excess of reducing power to drive the reaction against the laws of energy. The reduction of CO_2_ to formaldehyde via formate requires NADH while the reduction of formaldehyde to methanol requires PQQH_2_. This exigency of reducing energy equivalent is compensated by the endogenous reductant source (PHB) of the cells, which is explained in “Biological Production of Methanol From Methane and Carbon Dioxide” section.

## Products Synthesized by Methanotrophs

Wild type methanotrophs possess an inherent potential to synthesize a wide-range of products (Table 1). Molecules like ectoine (*Methylomicrobium alcaliphilum*, *Methylobacter marinus, Methylomicrobium kenyense, Methylobacter modestohalophilus*); hydroxyectoine (*Methylomicrobium alcaliphilum*); sucrose (*Methylomicrobium alcaliphilum, Methylomicrobium buryatense*, *Methylobacter marinus*); glutamate (*Methylobacter alcaliphilus, Methylomicrobium buryatense, Methylobacter modestohalophilus*); 5-oxoproline (*Methylobacter alcaliphilus*, *Methylobacter modestohalophilus*) are known as “compatible solutes.” These are secreted by halotolerant and halophilic methanotrophs in response to high salinity in the environment in order to balance the turgor pressure and minimize water-loss across the cell membrane (Khmelenina et al., 1999). They have applications in pharmaceutical industries as stabilizers for nucleic acids, DNA-protein complexes and enzymes. Methanotrophs are also able to produce extracellular polysaccharide (EPS) (*Methylomicrobium alcaliphilum* and methanotrophs enriched from soil/compost) as a defense mechanism against heat, desiccation, predation and other adverse conditions (Cantera et al., 2018). EPS finds application in textile, pharmaceutical and food industries owing to their adhesive and colloid-like properties.

**TABLE 1:** Products synthesized by methanotrophs.

Product	Strain	Substrate	Yield/Titer	References
Methanol	*Methylocella tundrae*	Methane-air mixture (50:50)	5.18 mM	Mardina et al., 2016
	*Methylosinus sporium*	Methane-air mixture (50:50)	5.80 mM	Patel et al., 2016c
	*Methylocystis bryophila*	Methane-air mixture (50:50)	4.63 mM	Patel et al., 2016b
	*Methylocystis bryophila*	Methane-air mixture (30:70)	52.9 mM	Patel et al., 2020c
	*Methylosinus trichosporium* IMV 3011	CO_2_-air mixture (50:40)	0.004 μmol/mg dry cell weight	Xin et al., 2007
	*Methylosinus sporium*	CO_2_-air mixture (30:70)	0.33 mM	Patel et al., 2016a
Ectoine	*Methylomicrobium alcaliphilum* ML1	Methane-air mixture (50:50)	230 mg g biomass^–1^	Reshetnikov et al., 2011
	*Methylobacter marinus* 7C	Methane-air mixture (50:50)	60 mg g biomass^–1^	Reshetnikov et al., 2011
	*Methylomicrobium kenyense* AMO1^*T*^	Methane-air mixture (50:50)	70 mg g biomass^–1^	Reshetnikov et al., 2011
	Methanotrophic bacterial consortium	Methane-air mixture (10:90)	0.42-1.33 mg g biomass^–1^	Stȩpniewska et al., 2014
	*Methylomicrobium alcaliphilum* 20Z	Methane-air mixture (50:50)	79.7–94.2 mg g biomass^–1^	Cantera et al., 2018
	*Methylomicrobium alcaliphilum* 20Z	Methane-air mixture (50:50)	12.3 mg g biomass^–1^	Mustakhimov et al., 2019
Hydroxyectoine	*Methylomicrobium alcaliphilum* 20Z	Methane-air mixture (50:50)	13 mg g biomass^–1^	Cantera et al., 2018
	*Methylomicrobium alcaliphilum* 20Z	Methane-air mixture (50:50)	19.7 mg g biomass^–1^	Mustakhimov et al., 2019
Sucrose	*Methylomicrobium alcaliphilum, Methylomicrobium buryatense*, *Methylobacter marinus*	Methane-air mixture (50:50)	30–60 mg/g dry cells	Khmelenina et al., 2010
Glutamate	*Methylomicrobium alcaliphilum, Methylomicrobium buryatense, Methylobacter marinus*	Methane-air mixture (50:50)	25–55 mg/g dry cells	Khmelenina et al., 2010
Single cell protein	*Methylococcus capsulatus*	Methane: O_2_: CO_2_ mixture (60:30:10)	52.52% of dry cell weight	Rasouli et al., 2018
	*Methylomonas* spp. and *Methylophilus* spp.	Methane-O_2_ mixture (40:60)	>50% of dry cell weight	Tsapekos et al., 2020
	*Methylomonas* sp. and *Methylocystis* sp.	Methane-O_2_ mixture	51% of the dry weight	Valverde-Pérez et al., 2020
Poly-β-hydroxybutyrate (PHB)	*Methylocystis parvus* OBBP	Methane-O_2_ mixture (50:50)	60% of dry weight	Rostkowski et al., 2013
	*Methylosinus trichosporium* OB3b	Methane-O_2_ mixture (50:50)	29% of dry weight	Rostkowski et al., 2013
	*Methylocystis hirsuta*	Methane-air mixture (4:96)	34.6 % of dry weight	García-Pérez et al., 2018
	*Methylocystis hirsuta*	Methanol: ethanol mixture (1:1)	85% of dry weight	Ghoddosi et al., 2019
Lipids	*Methylosphaera hansonii*	Methane-air mixture (50:50)	57% of dry cell weight	Schouten et al., 2000
	*Methylomicrobium buryatense*	Methane-air mixture (20:80)	10% of dry cell weight	Dong et al., 2017
	*Methylococcus capsulatus*	Methane: O_2_: CO_2_ mixture (60:30:10)	21.82% of dry cell weight	Rasouli et al., 2018
Extracellular polysaccharides	*Methylomicrobium alcaliphilum* 20Z	Methane-air mixture (50:50)	2.6 g L culture broth^–1^	Cantera et al., 2018
5-Oxoproline	*Methylobacter modestohalophilus* 10S	Methane-air mixture (50:50)	178 nmol (mg DCW)^–1^	Khmelenina et al., 1999
	*Methylobacter alcaliphilus* 20Z	Methane-air mixture (50:50)	248 nmol (mg DCW)^–1^	Khmelenina et al., 1999
Products obtained from genetically engineered methanotrophs				
Lactate	*Methylomicrobium buryatense*	Methane-air mixture (20:80)	0.8 g lactate/L	Henard et al., 2016
Astaxanthin	*Methylomonas* sp. 16a	Methane-air mixture (25:75)	80% of total carotenoids	Tao et al., 2007
	*Methylomonas* sp. 16a	Methane-air mixture (25:75)	95% of total carotenoids	Rick and Kelly, 2012

Methanotrophs also synthesize poly-β-hydroxybutyrate (PHB) (*Methylosinus trichosporium, Methylocystis parvus, Methylocystis hirsuta*), a potential replacement of conventional plastic owing to its biocompatible and biodegradable characteristics (Rodríguez et al., 2020). Single cell proteins, a major methanotrophic-product (*Methylococcus capsulatus, Methylomonas* sp., *Methylocystis* sp.), can be used as a prospective substitute for traditional protein sources viz., fishmeal and soymeal (Valverde-Pérez et al., 2020). Lipid molecules derived from methanotrophs (*Methylomicrobium buryatense, Methylococcus capsulatus*) can be explored as futuristic biofuel (Dong et al., 2017).

Studies have reported engineered (heterologous gene expression) methanotrophic strains to synthesize products beyond their innate metabolic potential. *Methylomicrobium buryatense* (Henard et al., 2016) and *Methylomicrobium alcaliphilum* (Henard et al., 2018) have been genetically engineered to produce lactate, which finds application in the production of bioplastics (from lactic acid polymers), cheese and yogurt, dermatological drugs etc. *Methylomonas* sp. 16a has been engineered to produce astaxanthin (Tao et al., 2007; Rick and Kelly, 2012), a carotenoid pigment with huge commercial significance.

Methanotrophs synthesize a wide-range of products (Table 1) using cheap and wasteful carbon-based substrates (CH_4_ and/or CO_2_) unlike microorganisms utilizing cost-intensive carbon sources like glucose, fructose, xylose etc. These features render methanotroph-based gas fermentation processes economical and sustainable.

## Biological Production of Methanol From Methane and Carbon Dioxide

Methanol is a clean-burning fuel with high specific-energy ratio, flame-speed and octane number; and low combustion temperature and volatility. As has been already discussed, methanotrophs utilize methane, which is subsequently oxidized to methanol, formaldehyde, formate, and finally CO_2_ (Figure 1). To achieve direct production of methanol from methane, the subsequent biochemical pathway leading to methanol oxidation needs to be blocked. This is often accomplished by adding MDH inhibitors in the medium. Several studies have reported various irreversible MDH inhibitors for directly producing methanol from methane, viz., cyclopropanol, phosphate, EDTA, MgCl_2_, NaCl, and NH_4_Cl (Takeguchi et al., 1997; Yoo et al., 2015; Patel et al., 2016c,b). However, the activity of MMO is dependent on NADH availability, and inhibition of methanol oxidation by MDH inhibitors results in the depletion of NADH. Hence, to accomplish uninterrupted oxidation of methane to methanol, it is essential to produce NADH by feeding formate to the culture medium (Xin et al., 2004b). Formate is added as a source of reducing power, which assists in the regeneration of NADH- the co-factor for methanol synthesis. However, the use of MDH inhibitors and formate is cost-intensive. Moreover, the supplementation of MDH inhibitors blocks the metabolic pathway in the methanotrophic strain. This affects the viability and biochemical and physiological functions of the culture. Consequently, it reduces the quantity and quality of the biomass produced. These factors lead to low methanol titer and also exclude the possibility of applying biomass-recycling strategy to this process.

Taking these drawbacks into consideration, it is imperative to look for alternative substrates or metabolic pathways which can be exploited to produce methanol using methanotrophs. The limitations associated with biological production of methanol from methane can be overcome by utilizing CO_2_ as the substrate for some methanotrophic species (Xin et al., 2007; Patel et al., 2016a). This is done in a two-stage process, where methane is utilized as carbon substrate during the first-stage to produce biomass, and CO_2_ is reduced by the generated biomass in the second-stage to produce methanol (Xin et al., 2007). CO_2_, an end-product of general methanotrophic metabolism (first-stage), tends to shift the reaction equilibrium in the backward direction if fed in excess to the cells (second-stage). Methanotrophic biomass act as biocatalyst for the reduction of CO_2_ to methanol. The reduction of CO_2_ to methanol is the reverse reaction of the oxidation of methane to CO_2_ (Figure 1). However, MMO lacks the ability to catalyze the reverse reaction (reduction of methanol to methane). Therefore, the end-product of the CO_2_ reduction pathway is methanol, which gets secreted extracellularly into the medium (Xin et al., 2004a, 2007). The reverse reaction requires excess of reducing equivalents, in the form of NADH, to drive the reaction against the laws of energy. Cells in general produce intracellular NADH. However, supply of endogenous NADH is limited and hence may be fed exogenously to the medium for uninterrupted progression of the batch; but this again adds to the cost of production. However, methanotrophs have an added advantage as they can produce PHB (Ghoddosi et al., 2019). PHB is also a source of intracellular reducing equivalents, in addition to NADH. β-hydroxybutyrate dehydrogenase, a NAD+-linked enzyme, catalyzes the decomposition of PHB to acetoacetic acid, and this subsequently generates reducing equivalents. Hence, the endogenously stored PHB in methanotrophs, plays a crucial role in accomplishing the reduction of CO_2_ to methanol.

Xin et al. (2007) reported the production of 0.004 μmol methanol/mg (DCW) through reduction of CO_2_ using *Methylosinus trichosporium* as a biocatalyst. The study also reports that cells having 38.6% PHB content showed highest methanol titers. Patel et al. (2016a) reported the production of 0.33 mM methanol using *Methylosinus sporium* from 30% CO_2_ feed. These are few studies which demonstrate methanol production from CO_2_. Although, there are reports suggesting CO_2_ assimilation in Type X and Verrumicrobial methanotrophs toward biomass formation, however, there is no evidence in literature regarding these species converting CO_2_ into methanol.

Authors have mainly demonstrated methanol production in batch and repeated-batch modes. Duan et al. (2011) has reported the production of 1.1 g/l methanol from 50% methane under optimum conditions in batch mode, using *M. trichosporium* OB3b. Patel et al. (2020c) reported the production of 30.9 mmol/l methanol from 30% methane using free-cells of *Methylocystis bryophila* under repeated-batch mode. Methanol production was reported to improve upto 52.9 mmol/l through covalent immobilization of cells on coconut coir (Patel et al., 2020c). Different studies have also reported improved methanol production through cell encapsulation in alginate and silica-gel (Patel et al., 2018), immobilization in polyvinyl alcohol (Patel et al., 2020b) and chemically modified chitosan (Patel et al., 2020a). Few key studies on methanol production using methanotrophs are listed in Table 1. Despite of the strategies involved, the processes are limited by low methanol titer and productivity. Yu et al. (2009) reported that methanol is toxic to *M. trichosporium* at concentrations above 3.0 g/l. This report illustrates that methanotrophs are able to tolerate a very low concentration of methanol and hence, suffer from end-product toxicity, leading to lower methanol titer and productivity.

## Mass Transfer and Solubility Limitations in Methanotroph Based Gas Fermentation

Common challenges associated with gas fermentation systems are gas-to-liquid mass transfer limitations and lower solubility of the gaseous substrates. Researchers have outlined the role of certain physico-chemical parameters like partial pressure, and extrinsic approaches like reactor configurations and mass transfer vectors to address these limitations.

Bioreactors help in achieving higher gas-to-liquid mass transfer rates as compared to other culture systems, facilitating easier and higher uptake of gases by the cells. Bubble column reactors have been predominantly used for cultivation systems where the objective is to replace mechanically driven stirring and to reduce the shear-stress on the cells. They are suitable for application when the requirement is to maintain directional flow and circulation, efficient mass transfer and heat transfer, especially while working with gas fermentation systems. Their application has mostly been reported for PHB production by methanotrophs (Ghoddosi et al., 2019; Rodríguez et al., 2020). Vertical tubular loop bioreactors have also been reported for gas fermentation. These reactors are characterized by defined direction of fluid circulation, often facilitated by a pump in gas-liquid based reactors and a propeller in fluidized bed reactors (Rahnama et al., 2012). Sheets et al. (2017) reported the production of methanol in a trickle-bed reactor (TBR) stuffed with ceramic beads. TBRs enhance air and methane mass transfer rates from the reactor headspace to the cells suspended in the medium. This study reported two-times greater oxygen mass transfer in the ceramic beads-stuffed reactor compared to the reactor without the bead-stuffing. The rate of methane oxidation was fourfold greater in the TBR as compared to shake-flask cultures. Volumetric mass-transfer coefficient (K_L_a) is a major metric for the performance evaluation of different reactor configurations. Airlift bioreactors have also been reported for high K_L_a values of upto 97.2 h^–1^ (Ghaz-Jahanian et al., 2018). Authors have reported the application of membrane biofilm reactors (MBR) for methanotroph-based bioremediation. MBRs overcome diffusional limitations by promoting high mass-transfer rates of sparingly soluble gaseous substrates to the biomass. MBRs have been reported for denitrification (Lee et al., 2019) and removal of perchlorate (Wu et al., 2019), chromate (Luo et al., 2019), vanadate (Wang et al., 2019), selenate (Shi et al., 2020), etc. from contaminated wastewater and groundwater using methanotrophs.

The problem of low solubility of gases in the liquid-phase in gas fermentation systems has been overcome by incorporating various modifications in the commonly used reactor configurations. Studies have reported the addition of mass transfer vector, an organic phase, which has high affinity for the gaseous substrate and eventually improves the gas hold-up in the liquid media and consequent increased availability of the gaseous substrate to the microbial cells (Rocha-Rios et al., 2010; Zúñiga et al., 2011). Ten percent silicone oil has been prevalently added as a “methane-vector” to the aqueous medium to demonstrate “two-phase partition bioreactors (TPPB).” Rocha-Rios et al. (2010) and Zúñiga et al. (2011) have reported upto 33–45 and 700% increase in methane-degradation, respectively, using TPPBs. Supplementary Table S1 summarizes different reactor configurations used for methanotrophic fermentations.

Partial pressure inside the reactor plays a crucial role in gas fermentation systems. The composition of the gas phase might vary depending upon the source, resulting in varying partial pressures. Timmers et al. (2015) reported that the growth of methanotrophs is faster at increased CH_4_ partial pressures, as this is associated with highly negative Gibb’s free energy change. While high partial pressures do result in increased solubility of the gases, they might interfere with the growth or product formation metabolism resulting from the interaction of the gaseous substrate with the key enzymes (Hurst and Lewis, 2010). In a study outlined by Soni et al. (1998), with pressure variation between 10 and 50 psi, *Methylomonas albus* showed a fourfold increase in methane uptake and 40% increase in biomass concentration at 20 psi, whereas higher pressure inhibited growth. Therefore, optimization of partial pressure is essential to maintain the balance between the solubility of gases and growth/product formation within the reactor.

## Conclusion and Future Perspectives

Methanotrophs are excellent candidates for sequestration of harmful C1 compounds (CH_4_ and CO_2_). They are cellular factories for synthesis of a wide-range of value-added products. Amongst them, methanol has drawn significant attention owing to its potential applicability as a biofuel. Although, methanotrophs can synthesize methanol directly from methane, the associated limitations have driven the researchers to look for alternate pathways. Production of methanol from the methanotrophic reduction of CO_2_ appears to be a promising alternative in this regard. The current state-of-the-art technology for the production of methanotrophic high-value products, is constrained by low product titer and productivity. As far as methanol production is concerned, limitations associated with product-toxicity are very challenging. Integration of the process with continuous/intermittent methanol recovery system may assist in overcoming product toxicity and improve its titer and productivity. Regardless of several reactor configurations outlined by researchers to deal with gas fermentation associated challenges, the technology stands at its “nascent stage of development.” Hence, it becomes imperative to conduct detailed in-depth studies aiming at the development of process-engineering and design strategies for sustainable synthesis of methanotrophic high-value products on a commercial-scale. GSMM may be applied to evaluate different methanotrophic species *in silico* to predict their yields, rates of production and consumption, directed toward improved production of high-value products, without performing time- and labor-consuming experiments.

## Author Contributions

KS collected the data. KS and GG wrote the manuscript. DD did conceptualization, manuscript reviewing, and editing. All authors contributed to the article and approved the submitted version.

## Conflict of Interest

The authors declare that the research was conducted in the absence of any commercial or financial relationships that could be construed as a potential conflict of interest.

## Footnotes

1 https://www.globalcarbonproject.org/carbonbudget/

2 https://www.globalmethane.org/about/methane.aspx

3 https://www.esrl.noaa.gov/gmd/ccgg/trends/gl_data.html; www.esrl.noaa.gov/gmd/ccgg/trends_ch4/

4 https://www.epa.gov/ghgemissions/understanding-global-warming-potentials
