# Supplementary material for: Biotransformation of Methane and Carbon Dioxide Into High-Value Products by Methanotrophs: Current State of Art and Future Prospects
Source: Front Microbiol. 2021 Mar 10;12:636486. doi: 10.3389/fmicb.2021.636486 (PMC7987672; doi:10.3389/fmicb.2021.636486)
Supplement: Supplementary file 1 [file Table_1.DOCX]

**Supplementary Material**

**Table S1: Reactor configurations for methanotroph-based gas fermentation**

| Methanotrophic species | Extrinsic modifications/features | Product/process | Performance indicator | Reference |
| --- | --- | --- | --- | --- |
| Trickle-bed reactor | | | | |
| *Methylocaldum* sp. 14B | Packed with ceramic beads | Methanol:  0.9 g/l/d;  Methane removal: 0.54 ± 0.03 mmol/h | K_L_a (O_2_) =9.54 ± 0.86 h^-1^ | Sheets et al., 2017 |
| Two-phase partition bioreactor | | | | |
| Methanotrophic consortium from activated sludge | Modified stirred tank reactor, with an organic phase/methane vector (silicone oil) to improve methane-availability to the cells. | CH_4_ removal= 16.9 ± 0.5 × 10^3^ g g^−1^ biomass h^−1^ (at 800 rpm, 10% silicone oil). | K_L_a (O_2_) =0.011 s^-1^ (at 500 rpm and 10% silicone oil). | Rocha‐Rios et al., 2010 |
| Methanotrophic consortium from activated sludge | Modified stirred tank and trickle bed reactor, with an organic phase/methane vector (10 % silicone oil) to improve methane-availability to the cells. | Methane degradation:  106 ± 7 g m^−3^_reactor_ h^−1^ (Stirred tank);  51 ± 7 g m^−3^_reactor_ h^−1^ (Trickle-bed) | - | Rocha-Rios et al., 2009 |
| *Methylobacterium*  *organophilum* CZ-2 | Modified stirred tank reactor, with an organic phase/methane vector (10% silicone oil) to improve methane-availability to the cells. | PHB: 38% (w w^−1^).  Methane degradation: 36 mg_CH4_ g^−1^_x_ h^−1^. | - | Zúñiga et al., 2011 |
| Internal loop airlift reactor | | | | |
| Methanotrophic consortium from activated sludge | Equipped with gas recirculation, and addition of methane vector (solid polymer, Desmopan). | Methane removal: 19-22 g m^−3^ h^−1^ | K_L_a (O_2_) =0.065 s^-1^ (at 10% Desmopan and 1 vvm gas recirculation rate) | Rocha‐Rios et al., 2011 |
| External-loop airlift bioreactor | | | | |
| Methanotrophic strains (AS1 and AS2) isolated from activated sludge | Equipped with a methane transfer chamber. | Methanol:  1600 mg/l | K_L_a (O_2_) _reactor_ = 97.2 h^−1^; K_L_a (CH_4_) _transfer chamber_ = 70.8 h^−1^. | Ghaz-Jahanian et al., 2018 |
| Bubble column bioreactor | | | | |
| *Methylocystis hirsuta* | Coupled with internal gas re-circulation. | PHB: 14.5 ± 2.9% (mg mg^-1^ TSS x 100).  Methane degradation: 41 g m^-3^ h^-1^ (approx.). | - | Rodríguez et al., 2020 |
| *Methylocystis hirsuta* | Equipped with internal gas-recycling  (at the rate of 0.50 m^3^_gas_ m^−3^_reactor_ min^−1^) | PHB: 34.6 ± 2.5%.  Methane degradation: 35.2 ± 0.4 g m^−3^ h^−1^. | - | García-Pérez et al., 2018 |
| *Methylocystis hirsuta* | - | PHB: 73.4% (w/w) of DCW | - | Ghoddosi et al., 2019 |
| *Methylocystis hirsuta* | - | PHB: 42.5% (w/w) of DCW | - | Rahnama et al., 2012 |
| Forced-liquid vertical tubular loop bioreactor | | | | |
| *Methylocystis hirsuta* | - | PHB: 51.6% (w/w) of DCW | - | Rahnama et al., 2012 |
| Stirred tank reactor | | | | |
| *Methylomicrobium alcaliphilum* 20Z | - | Ectoine: 37.4 mg/g biomass.  Methane degradation: 24.5 g m^-3^ h^-1^. | - | Cantera et al., 2017 |
